# Effect of Cross‐Linked Hyaluronate Scaffold on Cartilage Repair: An *In Vivo* Study

**DOI:** 10.1111/os.12508

**Published:** 2019-08-05

**Authors:** Shi‐peng Xiao, Lian‐sheng Tang, Jian‐ying Chen, Zhong‐tao Li, Guang‐hui Cheng, Qian‐qian Chen, Sheng‐hou Liu, Wen‐guang Liu

**Affiliations:** ^1^ Department of Joint Surgery and Sports Medicine The Second Hospital of Shandong University Jinan China; ^2^ Key Laboratory of Biopharmaceuticals of Shandong Province Shandong Academy of Pharmaceutical Sciences Jinan China; ^3^ Key Laboratory of Mucosal and Transdermal Drug Delivery Technology in Shandong Province Shandong Freda Pharmaceutical Group Jinan China; ^4^ Sehandong Provincial Key Laboratory of Network Based Intelligent Computing School of Information Science and Engineering, University of Jinan Jinan China; ^5^ Central Research Laboratory The Second Hospital of Shandong University Jinan China

**Keywords:** Biomechanical, Cartilage repair, Cross‐linked hyaluronate scaffold, Histology, Tissue engineering

## Abstract

**Objective:**

To determine the safety and effectiveness of a cross‐linked sodium hyaluronate (CHA) scaffold in cartilage repair.

**Methods:**

Physicochemical properties of the scaffold were determined. The safety and effectiveness of the scaffold for cartilage repair were evaluated in a minipig model of a full‐thickness cartilage defect with microfracture surgery. Postoperative observation and hematological examination were used to evaluate the safety of the CHA scaffold implantation. Pathological examination as well as biomechanical testing, including Young's modulus, stress relaxation time, and creep time, were conducted at 6 and 12 months postsurgery to assess the effectiveness of the scaffold for cartilage repair. Furthermore, type II collagen and glycosaminoglycan content were determined to confirm the influence of the scaffold in the damaged cartilage tissue.

**Results:**

The results showed that the routine hematological indexes of the experimental animals were within the normal physiological ranges, which confirmed the safety of CHA scaffold implantation. Based on macroscopic observation, it was evident that repair of the defective cartilage in the animal knee joint began during the 6 months postoperation and was gradually enhanced from the central to the surrounding region. The repair smoothness and color of the 12‐month cartilage samples from the operation area were better than those of the 6‐month samples, and the results for the CHA scaffold implantation group were better than the control group. Greater cell degeneration and degeneration of the adjacent cartilage was found in the implantation group compared with the control group at both 6 and 12 months postoperation, evaluated by O'Driscoll Articular Cartilage Histology Scoring. Implantation with the CHA scaffold matrix promoted cartilage repair and improved its compression capacity. The type II collagen level in the CHA scaffold implantation group tended to be higher than that in the control group at 6 months (2.33 ± 1.50 *vs* 1.68 ± 0.56) and 12 months postsurgery (3.37 ± 1.70 *vs* 2.06 ± 0.63). The GAG content in the cartilage of the control group was significantly lower than that of the experimental group (2.17 ± 0.43 *vs* 3.64 ± 1.17, *P* = 0.002 at 6 months and 2.27 ± 0.38 *vs* 4.12 ± 1.02, *P* = 0.002 at 12 months). Type II collagen and glycosaminoglycan content also demonstrated that CHA was beneficial for the accumulation of both these vital substances in the cartilage tissue.

**Conclusions:**

The CHA scaffold displayed the ability to promote cartilage repair when applied in microfracture surgery, which makes it a promising material for application in the area of cartilage tissue engineering.

## Introduction

Hyaluronic acid (HA) is a glycosaminoglycan consisting of repeating disaccharide units of (1‐β‐4) D‐glucuronic acid and (1‐β‐3) N‐acetyl‐D‐glucosamine^1^. It is an important component of the extracellular matrix in tissues, including skin, corpus vitreum, synovial fluid, and cartilage, having unique physicochemical properties and biological functions^2^.

Commercial HA is supplied in the form of sodium salt (sodium hyaluronate [SH]) prepared by microbial fermentation or animal‐tissue extraction, and is now widely used in the areas of food, cosmetics, and medicine. HA as a substrate for the growth of chondrocytes is an important component of the cartilage matrix, which can promote the metabolism of chondrocytes and stimulate cartilage matrix formation as well as maintaining chondrocyte phenotype^3^. It is one of the most suitable materials to use in construction of a chondrocyte scaffold.

Raw HA display hygroscopicity and high water solubility because of their polymers with long linear chains. They are readily distributed and degraded by inherent enzymes or free radicals in bodily tissues. Injectable HA in its natural form lasts only 1–2 days when applied to local body tissue^4^. The mechanical strength of non‐cross‐linked HA cannot meet the requirement of a scaffold for tissue engineering. A stable network of the polymer is formed by cross‐linking. Biodegradable and porous material can be prepared by controlling the degree of cross‐linking and the process parameters, which would generate a potential scaffold for tissue engineering of cartilage. An injectable gel of a cross‐linked HA (CHA) with 1,4‐butanediol diglycidyl ether (BDDE) as a cross‐linker was used to treat osteoarthritis (OA) and soft‐tissue defects and for filling dermal tissue. The efficacy and safety of these products have been confirmed in clinical trials^5^, ^6^. However, the efficacy of CHA in tissue engineering has, to date, not been clarified.

In the present paper, CHA scaffold samples were prepared using a method of double freeze‐drying, with BDDE as a cross‐linker. Our study was conducted to assess the efficacy of the CHA scaffold as a newly developed implant in cartilage repair. Therefore, we needed to clarify three aspects of the scaffold implantation: (i) because safety is the most important factor for implants, the safety of the CHA scaffold was assessed by determining the physicochemical properties of the scaffold; (ii) the effect of the scaffold on cartilage repair was evaluated in a minipig full‐thickness cartilage defect model with microfracture surgery; and (iii) cartilage‐related component detection and pathological examination of the repaired cartilage were used to confirm the promotive effect of the CHA scaffold in cartilage repair at the microscopic and molecular levels. Therefore, the present study will provide the basis for the application of the CHA scaffold in the area of cartilage engineering.

## Materials and Methods

### *Preparation of Cross‐Linked Sodium Hyaluronate Scaffold*

The CHA scaffold was obtained from our previous study, and was generated from SH with BDDE as cross‐linker using a method with a double freeze‐drying process^7^. Small CHA scaffold discs of 5‐mm diameter were made using a punch for the following surgery.

### *Animal Model of Full‐Thickness Cartilage Defect*

A total of 12 common grade minipigs weighing 20 ± 2 kg and aged 4–5 months were provided by Shanghai Nanhui Laogang Huaxing Special Animal Breeding Center (Shanghai, China).

The experimental minipigs were anesthetized using 1% pentobarbital sodium solution, were fixed, and routine skin disinfection was performed. A skin incision of 2–3 cm was made in the left and right knees to expose the joints. The femoral condyle was accessed by dislocating the patella and inclining the knee joint ligament, whereupon a cylindrical cartilage defect of 5‐mm diameter was created in the femoral condyle. During surgery, the subchondral bone was debrided carefully to avoid bleeding and to ensure a standard‐size defect. Subsequently, microfracture holes were made in the defect using a 90° microfracture hand cone according to the procedure described by Schneider *et al*.^8^ The holes were at a distance of approximately 2–3 mm apart and a depth of approximately 3–4 mm^8^.

Following surgery in the CHA scaffold implantation group, the right knees of six minipigs were implanted with the CHA scaffold, the normal structure of the joints was restored and the wound was closed. The left knee joints of the other six minipigs acted as controls, which received no implant, with only the structure restored surgically and the wound closed. In addition, the normal group referred to the undamaged part adjacent to the cartilage defect region.

### *Cross‐Linked Sodium Hyaluronate Scaffold Implantation*

The right knee joint cartilage defect was coated with a thin layer of fibrin glue according to the manufacturer's instructions on the bottom of the defect and around the defective cartilage edge. The CHA scaffold disc was pressed into the defect, wetting by dripping saline, and pressed lightly using the surgical knife handle to complete the adhesive fixation. The patella and skin were reset to cover the defective area, and the bending action of the knee joint could further compact the implanted material. And then the skin and patellar was pushed aside to check if the implanted material was compacted tightly. Joint restoration and suture followed.

Subsequently, the animals were allowed to move freely and received standard animal feed and clean drinking water. The room temperature was maintained at 15–26°C. A daily UV disinfection was performed and a postoperative intramuscular injection of 5 × 10^8^ units of penicillin was made every morning and afternoon for 3 days to prevent postoperative infection.

### *Postoperative Observation*

An observation was performed outside the cage once daily for 7 days postoperation and subsequently once weekly, with a detailed recording of the appearance of signs, behavioral activities, salivation, respiratory conditions, urination, and administration of local reactions.

### *Hematological Examination*

Blood samples were taken from the veins of animals at 6 and 12 months postsurgery. The blood samples were collected using a Sysmex XT‐2000iv automatic hematology analyzer (Sysmex, XT‐2000iv, Japan), centrifuged at 1000 g for 15 min and serum biochemical detection was performed using an automatic biochemical analyzer (Hitachi, 7180, Japan).

### *Pathological Examination*

Macroscopic observation was conducted at 6 and 12 months postsurgery. Three animals from each group were used for the pathological examination at 6 months and the remaining three animals of each group at 12 months. The animals were anesthetized using 1% sodium pentobarbital solution, the common carotid arteries were removed, and the entire hind legs were removed to reveal the knee joint to observe the defect repair, including the degree of defect repair, surface roughness, and color change. With the surrounding normal cartilage integration, the border was clear. Synovium of joint proliferation was also detected.

Following macroscopic photography, the normal cartilage and subchondral bone of the defect repair site and adjacent undamaged sites were cut using an 8‐mm trephine. After the samples were taken, the remaining material was stored at −80°C. Defect‐centered cartilage surface tissue and subchondral bone of 1.5 cm was cut off and fixed in 10% neutral formalin for more than 24 h. This was decalcified with 10% EDTA decalcification solution for 30 days, dehydrated in an ethanol gradient and then xylene and embedded vertically in paraffin. Sections of 5 μm were cut and stained using safranin‐O according to the standard method. An O'Driscoll's Articular Cartilage Histological Score was calculated according to the reported criteria shown in Table 1, 9.

**Table 1:** O'Driscoll's articular cartilage histological score

Index	Score
Nature of the predominant tissue	
Cellular morphology	
Hyaline articular cartilage	4
Incompletely differentiated mesenchyme	2
Fibrous tissue or bone	0
Safranin‐O staining of the matrix	
Normal or nearly normal	3
Moderate	2
Slight	1
None	0
Structural characteristics	
Surface regularity	
Smooth and intact	3
Superficial horizontal lamination	2
Fissures −25% to 100% of the thickness	1
Severe disruption. Including fibrillation	0
Structural integrity	
Normal	2
Slight disruption. Including cysts	1
Severe disintegration	0
Thickness	
100% of normal adjacent cartilage	2
50%–100% of normal cartilage	1
0%–50% of normal cartilage	0
Freedom from cellular changes of degeneration	
Hypocellularity	
Normal cellularity	3
Slight hypocellularity	2
Moderate hypocellularity	1
Severe hypocellularity	0
Chondrocyte clustering	
No clusters	2
<25% of the cells	1
25%–100% of the cells	0
Freedom from degenerative changes in adjacent cartilage	
Normal cellularity, no clusters, normal staining	3
Normal cellularity, mild clusters, moderate staining	2
Mild or moderate hypocellularity, slight staining	1
Severe hypocellularity. Poor or no staining	0

### *Biomechanical Testing*

The repaired cartilage and normal cartilage samples frozen at −80°C were thawed at room temperature and an indentation test was performed. The Young's modulus (MPa), stress relaxation time (s), and creep time (s) were determined *in vitro* using an Instron materials testing machine (Instron, type 4302, UK) at room temperature. Specimens were kept moist using Ringer's solution and an ultrasound moistener. Vernier calipers were used to calculate the cross‐sectional area of the specimens. All data are given as the mean force value in each cross‐sectional area applied to the cartilage. All tests were performed using a 100‐N sensor and the displacement was controlled by the Instron machine. Preconditioning was performed in all tests.

For the Young's modulus test, the specimens were pulled continuously at a constant rate of 10 mm/min until a maximum stress of 2.5 MPa was attained. For the stress relaxation test, the specimens were rapidly extended at a rate of 125 mm/min from their natural state to a maximum stress of 2.5 MPa, followed by a relaxation time of 10 min. In the creep test, the specimens were rapidly extended at a rate of 10 mm/min from their natural state to a maximum stress of 2.5 MPa, and this stress level was maintained for 10 min. While maintaining the maximum stress level, the specimens continued to deform; this phenomenon is called “creep.” All data are the mean of at least three measurements and are expressed as the mean ± SD. Each factor was compared between the different groups using one‐way analysis of variance (ANOVA)^10^.

### *Type II Collagen Assay*

The repaired cartilage tissues were completely cut out using a 6‐mm diameter trephine, as were equal amounts of normal cartilage tissues. A small part of these tissues was used for the quantitative determination of type II collagen, while the remainder was used to determine the glycosaminoglycan content.

The cartilage samples were weighed and then lyophilized to obtain the dry weight to determine the amount of collagen in both the wet and dry tissues. A total of 1–2 mg of lyophilized sample was transferred to a centrifuge tube to which was added 0.5 mL pre‐chilled distilled water. After overnight incubation at 4°C, the sample was centrifuged at 8000 g for 3 min. The supernatant was discarded and the pellet resuspended in 0.5 mL 0.05 M Tris–HCl buffer (pH 7.5) and shaken overnight at 4°C. Following centrifugation at 8000 g for 3 min, the supernatant was transferred to a collection tube with 0.2 mL goat serum buffer. The precipitate generated was washed with 0.5 mL of pre‐cooled distilled water and 0.5 mL 0.05 M acetic acid was added for incubation at 4°C overnight. The suspension was centrifuged at 8000 g for 3 min and the supernatant was transferred to a collection tube. The combined supernatants were dialyzed in 0.1 M Tris‐0.15 M NaCl buffer (pH 7.5) and stored at −20°C.

The sediment obtained was digested using pepsin, until almost the entire tissue fragments were dissolved. This was followed by elastase digestion using 0.5 mL of 0.1 mg/mL pancreatic elastase solution. The supernatant was collected. The supernatants acquired from the pepsin and elastase digestions were combined for the determination of type II collagen content. To this was added 1/50 volume of 1M Tris base was added and the volume was adjusted to 2–5 mL with 0. 1 M Tris‐0.15 M NaCl. The collagen content was determined by ELISA assay using the Porcine collagen II (Col II) Elisa Kit (Elixir Canada Medicine Company, Hermes Criterion Biotechnology, Canada) according to the manufacturer's instructions.

### *Determination of Glycosaminoglycan Content*

Fresh cartilage samples were lyophilized and cut into small pieces. These were soaked in 95% alcohol for 2–3 h and then in acetone for 2 h and wrapped in filter paper to defat for 16 h in a Soxhlet apparatus. The degreased samples were dried at 80°C for 4 h and ground to a fine powder.

This fine powder was digested by incubation with 1 mL trypsin at 37°C for 24 h, 1 mL of papain at 37°C for 24 h, and 60% trichloroacetic acid at a final concentration of 10% at 4°C overnight, and the supernatant was collected. Three times the volume of ethanol was added to the supernatant, left for 24 h and centrifuged at 11 500 g for 30 min; the supernatant was discarded. The remaining freeze‐dried precipitate was crude GAG products, which was used to determine the GAG content.

Glycosaminoglycan and alcian blue rapidly generate a soluble GAG‐alcian blue complex. The light absorption of this complex is different from alcian blue. Therefore, the GAG content can be calculated by colorimetric determination of the level of the GAG–alcian blue complex. The sample GAG content was determined by ELISA assay using the Porcine glycosaminoglycan (GAG) Elisa Kit (Elixir Canada Medicine, Hermes Criterion Biotechnology, Canada) according to the manufacturer's instructions.

### *Statistical Analysis*

Statistical analysis was performed using the statistical software SPSS, version 16.0 (IBM SPSS, Chicago, US). Continuous data, including hematological factors, Young modulus, stress relaxation time, creep time, and type II collagen as well as GAG content were expressed as means ± standard deviation, and the statistical significance of those variables were evaluated with Student's *t*‐test and one‐way ANOVA. All tests were two‐sided and *P* < 0.05 was considered statistically significant. Selected significant differences between groups are highlighted in the Results section, with complete statistically significant differences reported in the tables and figures.

### *Ethical Approval*

All procedures performed involving animals in this study were in accordance with the ethical standards of the Second Hospital of Shandong University Research Committee.

## Results

### *Cross‐Linked Sodium Hyaluronate Scaffold Properties*

**Figure 1:**
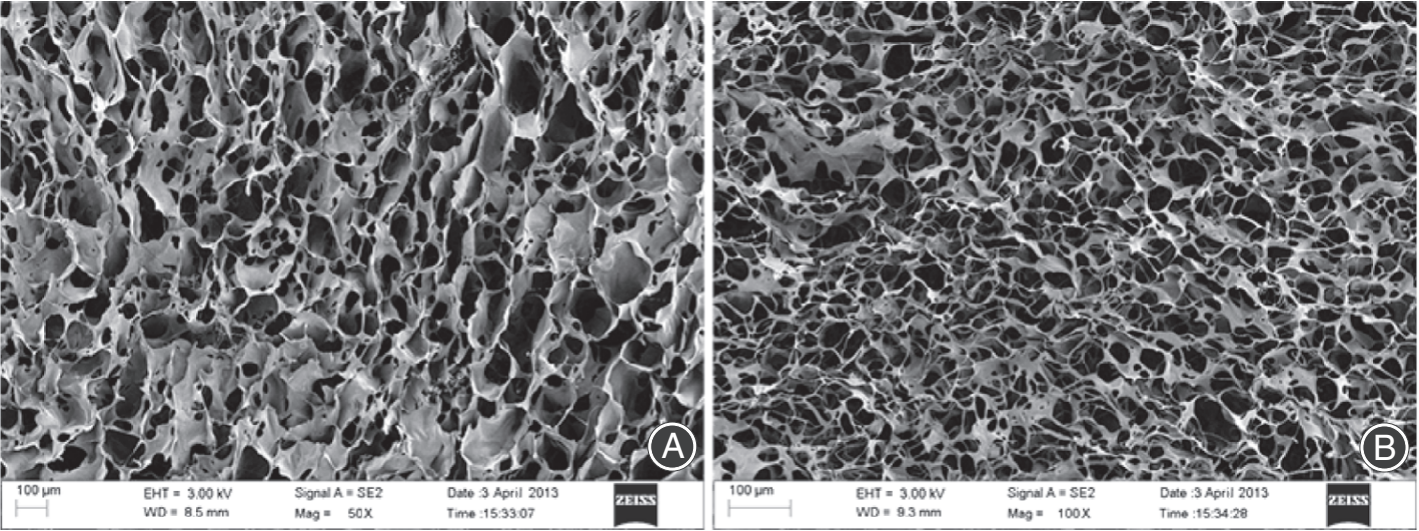
Scanning electron microscopy (SEM) morphology of cross‐linked sodium hyaluronate (CHA) scaffold. (A) Upper surface; the upper surface showed porous and network structures. (B) Lateral section; the lateral section showed a porous, sheet‐like tube network. The CHA scaffold had a porous spongy appearance with a pore size of 80–150 μm under SEM.

**Figure 2:**
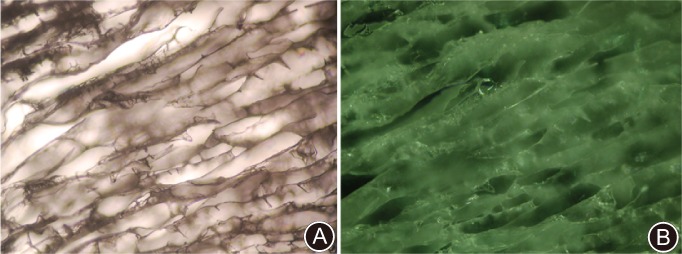
Morphology of cross‐linked sodium hyaluronate (CHA) by optical microscopy. (A) Transmitted light 100×. (B) Reflected light 100×. The inner structure of the scaffold showed an interconnected tubular network with wrinkled or smooth wall under optical microscopy.

**Figure 3:**
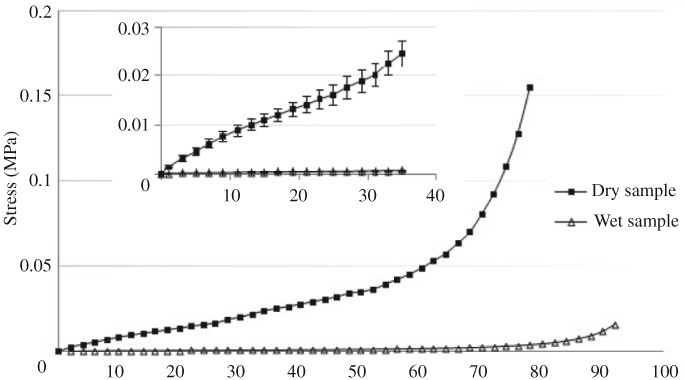
Stress–strain curve for dry sample and wet sample of CHA scaffold. A lower compression modulus of wet scaffold maintained in whole strain range suggested that inner pores and tubes of the scaffold were well interconnected.

Examination of the physicochemical properties showed the CHA scaffold to be of a porous spongy appearance with a pore size of 80–150 μm under scanning electron microscopy (Figs 1 and 2). The degree of modification (MD) of the CHA scaffold was 3.10% ± 0.27% (Table 2). The compression strength (CS) for the dry and wet samples was 14.80 ± 4.06 kPas and 0.5762 ± 0.11 kPas, respectively (Fig. 3), and the compressive modulus (CM) was 0.6009 ± 0.0837 kPas and 0.0156 ± 0.0042 kPas, respectively; the water absorption (WA) was 58.6% ± 3.5%, the expansion ratio (EX) 100.7% ± 4.8%, and the porosity (P) 96.5% ± 2.6%.

**Table 2:** Data of a cross‐linked sodium hyaluronate scaffold on physicochemical properties

Properties	Values (mean ± SD)
Degree of modification (*MD*, %)	3.10 ± 0.27
Compression strength (*CS*, kPa)	
Dry sample	14.80 ± 4.06
Wet sample	0.5762 ± 0.11
Compressive modulus (*CM*, kPa)	
Dry sample	0.6009 ± 0.0837
Wet sample	0.0156 ± 0.0042
Water absorption (*WA*, %)	58.6 ± 3.5
Expansion ratio (*EX*, %)	100.7 ± 5.8
Porosity (*P*, %)	96.5 ± 2.0

### *General Postoperative Observation*

The animals recovered from anesthesia 3–5 h postoperation. After 24 h, the animals could stand up, with left hindlimb claudication, while food and drinking water intake were generally normal. Within 15 days postoperation, the surgical sites had almost recovered, with shiny fur. Moreover, there was no secretion around the eyes, nose or ears, nor trauma or inflammation in other body parts. In addition, behavior and gait were nearly normal, without any obvious clinical adverse reactions being found.

### *Hematological Examination Postoperation*

The hematological factors were evaluated at 6 and 12 months postoperation to determine the influence of the surgery and the CHA scaffold implantation. Most of the results of the routine blood, hepatorenal function, and biochemical ion tests displayed no significant differences when comparing the CHA scaffold implantation group with the control group, except for some biochemical ions and lipid factors (Table 3). Furthermore, these routine hematological factors tested in all the enrolled animals were within the normal physiological ranges.

**Table 3:** Comparison of the hematological factors between the groups after surgery for 6 and 12 months (mean ± SD)

Variable	6 months post‐operation			12 months post‐operation		
	CHA scaffold implantation group (*n* = 6)	Control group (*n* = 6)	*P*	CHA scaffold implantation group (*n* = 6)	Control group (n = 6)	*P*
WBC	18.23 ± 5.10	19.69 ± 0.80	0.59	16.85 ± 2.45	14.70 ± 1.13	0.08
RBC	10.15 ± 0.44	9.65 ± 0.70	0.24	8.00 ± 0.44	8.16 ± 0.20	0.43
HGB	163.50 ± 5.36	158.50 ± 9.16	0.32	133.00 ± 7.92	140.50 ± 6.06	0.10
PLT	243.57 ± 170.94	241.40 ± 80.54	0.82	429.10 ± 67.97	325.60 ± 144.76	0.14
HCT	46.21 ± 3.91	45.93 ± 3.95	0.96	35.83 ± 2.05	37.68 ± 2.10	0.15
NEUT%	35.70 ± 8.44	35.63 ± 2.16	0.97	31.42 ± 2.42	36.70 ± 10.37	0.25
LYMPH%	56.65 ± 7.88	56.58 ± 3.45	0.92	42.95 ± 5.66	45.05 ± 7.43	0.59
MONO%	3.20 ± 2.05	2.92 ± 0.39	0.84	4.28 ± 0.97	3.42 ± 1.01	0.16
EO%	3.72 ± 1.41	4.18 ± 1.57	0.61	20.68 ± 7.27	14.50 ± 10.52	0.26
BASO%	0.73 ± 0.30	0.68 ± 0.15	0.64	0.67 ± 0.45	0.33 ± 0.12	0.11
PT	11.48 ± 0.19	11.67 ± 0.21	0.24	11.58 ± 0.17	11.40 ± 0.19	0.11
TT	13.75 ± 0.48	13.48 ± 0.95	0.67	13.90 ± 0.41	13.40 ± 0.58	0.12
ALT	78.83 ± 7.19	79.66 ± 12.46	0.70	71.43 ± 7.15	74.98 ± 4.44	0.33
AST	40.92 ± 5.73	38.00 ± 6.56	0.56	53.98 ± 8.83	53.13 ± 6.66	0.85
TP	70.85 ± 2.90	68.92 ± 3.47	0.27	67.68 ± 4.87	63.85 ± 5.39	0.23
ALB	43.28 ± 2.15	41.00 ± 2.79	0.24	40.45 ± 4.50	37.48 ± 4.41	0.28
TBIL	1.76 ± 0.81	0.99 ± 0.40	0.07	0.97 ± 0.24	1.34 ± 0.50	0.13
ALP	260.83 ± 164.27	285.8 ± 109.87	0.95	177.17 ± 46.35	163.33 ± 45.54	0.61
BUN	3.08 ± 0.56	2.48 ± 0.39	0.10	1.70 ± 0.15	1.73 ± 0.18	0.73
CREA	55.23 ± 3.81	60.58 ± 5.88	0.05	103.17 ± 0.36	92.22 ± 12.39	0.06
TC	2.32 ± 0.57	2.26 ± 0.11	0.59	2.20 ± 0.07	2.01 ± 0.14	0.02*
TG	0.33 ± 0.08	0.28 ± 0.04	0.12	0.45 ± 0.05	0.28 ± 0.12	0.01*
CK	279.17 ± 121.21	316.20 ± 35.03	0.35	1058.1 ± 222.33	755.17 ± 221.46	0.04*
K	5.55 ± 0.79	5.16 ± 0.39	0.43	3.37 ± 0.05	4.18 ± 1.21	0.13
Na	144.17 ± 1.60	145.80 ± 0.84	0.46	145.50 ± 0.55	144.00 ± 1.10	0.01*
Cl	96.50 ± 1.38	99.60 ± 0.89	0.002*	103.00 ± 0.63	102.17 ± 1.33	0.20

ALB, albumin; ALP, alkaline phosphatase; ALT, glutamic‐pyruvic transaminase; AST, glutamic oxalacetic transaminase; BASO, basophil; BUN, blood urea nitrogen; CHA, cross‐linked sodium hyaluronate; CK, creatine kinase; Cl, chlorinum; CREA, creatinine; EO, eosinophil; HCT, hematocrit; HGB, hemoglobin; K, Potassium; LYMPH, lymphocyte; MONO, mononuclear leucocyte; Na, sodium; NEUT, neutrophile granulocyte; PLT, platelets; PT, prothrombin time; RBC, red blood cell; TBIL, total bilirubin; TC, total cholesterol; TG, triglyceride; TP, total protein; TT, thrombin time; WBC, white blood cell

* *P*‐value of *P* < 0.05 was considered statistically significant.

### *Pathological Characteristics*

#### *Macroscopic Observation*

**Figure 4:**
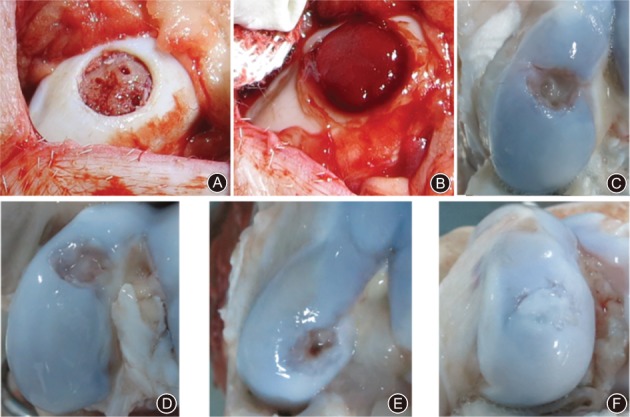
Macroscopic observation of the defected cartilage samples. (A) Microfracture model. (B) Cross‐linked sodium hyaluronate (CHA) scaffold implantation in microfracture site. (C) 6 months after microfracture surgery. Repair of the defective cartilage in the animal knee joint began during the 6 months postoperation and was gradually enhanced from the central to the surrounding region under macroscopic observation. However, the repaired surface of the defect was snatchy, and the junction between the defect and repaired areas was not clear. (D) 12 months after microfracture surgery. There was no significant improvement in the control group of the cartilage surface or margin compared to after 6 months postoperation. (E) 6 months after CHA scaffold implantation in the microfracture site. After 6 months, the cartilage defect was repaired in the CHA scaffold implantation group, and the junction of the defect and the repaired areas was clear and tight without any cracks. (F) 12 months after CHA scaffold implantation in microfracture site. The cartilage surface in the repair area of the CHA scaffold implantation knee was smooth and the color was similar to that of normal cartilage. There were no obvious boundaries between the normal and the repaired areas and the degree of repair had clearly improved compared with that at 6 months.

Repair of the defective cartilage in the animal knee joint began during the 6 months postoperation and was gradually enhanced from the central to the surrounding region under macroscopic observation. After 6 months, the cartilage defect was repaired in the CHA scaffold implantation group, and the junction of the defect and the repaired areas was clear and tight without any cracks, but the repaired area appeared slightly paler compared to the normal cartilage (Fig. 4). However, the repaired surface of the defect in the control group was snatchy, and the junction between the defect and repaired areas was not clear.

At 12 months postoperation, the cartilage surface in the repair area of the CHA scaffold implantation knee was smooth and the color was similar to that of normal cartilage. The margin of the repaired area was basically merged with the normal cartilage. There were no obvious boundaries between the normal and the repaired areas and the degree of repair had clearly improved compared with that at 6 months. By contrast, there was no significant improvement in the control group of the cartilage surface or margin after 12 months compared to after 6 months postoperation.

#### *Microscopic Examination*

**Figure 5:**
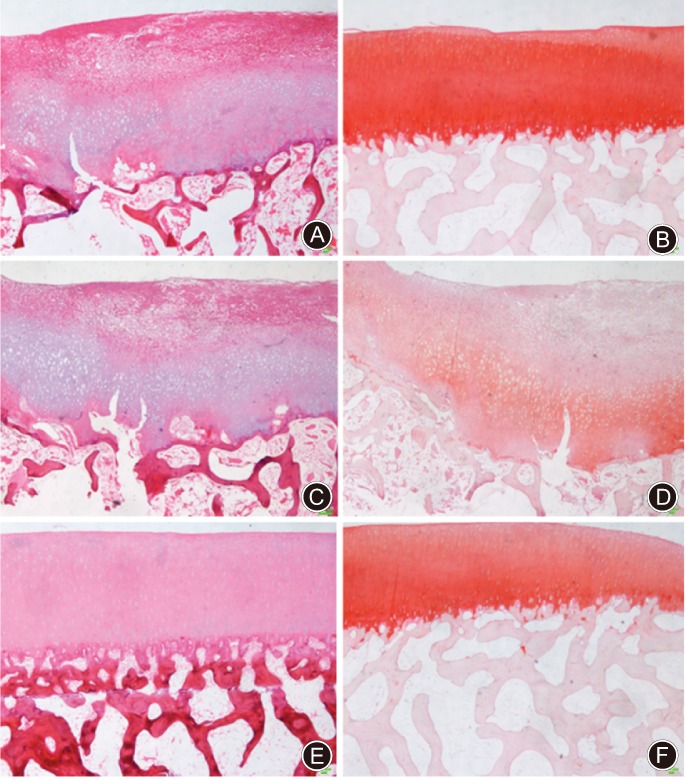
Microscopic observation and safranin O staining results of the defected cartilage samples. (A) Hematoxylin–eosin (HE) staining of repaired tissue from the cross‐linked sodium hyaluronate (CHA) scaffold implantation group (×4). (B) Safranin O staining of repaired tissue from the CHA scaffold implantation group (×4). (C) HE staining of repaired tissue from the microfracture group (×4). (D) Safranin O staining of repaired tissue from the microfracture group (×4). (E) HE staining of normal cartilage tissue (×4). (F) Safranin O staining of normal cartilage tissue (×4). HE staining showed a dark blue nucleus and a red matrix around the cartilage, with varying degrees of fibrous tissue repair. With safranin‐O staining, the superficial and even deep layers in the repaired cartilage tissue of the implantation group were lightly and unevenly dyed, whereas in the control group, this was more shallow and uneven.

Furthermore, microscopic examination of HE staining of tissues acquired at 6 and 12 months postsurgery showed that some chondrocytes were present on the cartilage surface of the CHA implanted animals. Moreover, HE staining showed a dark blue nucleus and a red matrix around the cartilage, with varying degrees of fibrous tissue repair (Fig. 5).

With safranin‐O staining, the superficial and even deep layers in the repaired cartilage tissue of the implantation group were lightly and unevenly dyed after 6 months, whereas in the control group, this was more shallow and uneven. Furthermore, the matrix of the repaired area of the implantation group was stained much deeper at 12 months postoperation, but in the control group, only the superficial layer of cartilage and even the middle layer displayed only slight staining and uneven coloring (Fig. 5).

#### *O'Driscoll Articular Cartilage Histology Scoring*

We applied the O'Driscoll Articular Cartilage Histology Scoring Criteria to evaluate the major histological types, structural features, cell degeneration, and degenerative changes of adjacent cartilage. The results of the composite scores of cartilage repair of the left and right knees are presented in Table 4. Greater cell degeneration and degeneration of the adjacent cartilage was found in the implantation group compared with the control group at both 6 and 12 months postoperation, suggesting that the CHA scaffold could reduce injury‐induced cartilage degeneration and might promote the cartilage defect repair ability.

**Table 4:** Comparison of the O'Driscoll histological grade between the groups

Variable	Score			
	CHA scaffold implantation group		Control group	
	6 months	12 months	6 months	12 months
Nature of the predominant tissue	2.7	2.8	2.2	2.4
Structural characteristics	2.4	2.7	2.0	2.5
Freedom from cellular changes of degeneration	1.3	1.5	1.0	1.3
Freedom from degenerative changes in adjacent cartilage	2.0	2.1	1.8	2.0
Total	8.4	9.1	7.0	8.2

CHA, cross‐linked sodium hyaluronate

### *Biomechanical Properties of the Repaired Cartilage*

**Figure 6:**
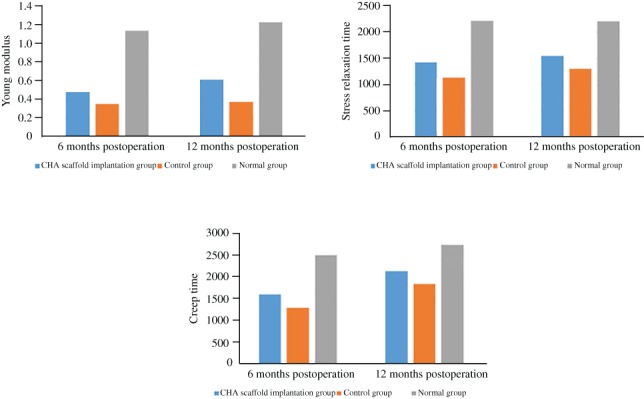
Biomechanical properties of the repaired cartilage between the groups. (A) Young modulus. (B) Stress relaxation time. (C) Creep time. Our results suggested that the mean repair area for the implanted scaffold was greater than that of the control group, the former being closer to the values of the normal area cartilage, indicating that cross‐linked sodium hyaluronate (CHA) scaffold cartilage implantation was better than the operation control regarding the biomechanical properties of the repaired cartilage.

The biomechanical properties was evaluated 6 and 12 months postoperation by Young modulus, stress‐relaxation time, and creep time. As shown in Table 5, there were statistically significant differences in the biomechanical properties among the groups at both at 6 months and 12 months postoperatively. For the scaffold implantation, control, and normal groups, the Young modulus was 0.48 ± 0.16, 0.35 ± 0.06, and 1.14 ± 0.16, respectively, at 6 months and 0.61 ± 0.17, 0.37 ± 0.13, and 1.23 ± 0.22, respectively, at 12 months; the stress relaxation time was 1448 ± 337, 1132 ± 257, and 2222 ± 217, respectively, at 6 months and 1544 ± 407, 1297 ± 70, and 2209 ± 461, respectively, at 12 months; the creep time was 1598 ± 314, 1297 ± 339, and 2504 ± 151, respectively, at 6 months and 2132 ± 213, 1838 ± 262, and 2738 ± 237, respectively, at 12 months. Our results suggested that the mean repair area for the implanted scaffold was greater (*P* < 0.001 for Young modulus, stress‐relaxation time, and creep time at 6 months postoperation and *P* < 0.001 for Young modulus, *P* = 0.015 for stress‐relaxation time, and *P* = 0.001 for creep time at 12 months postoperation) than that of the control group of the left knee repair area (Fig. 6), the former being closer to the values of the normal area cartilage, indicating that CHA scaffold cartilage implantation was better than the operation control regarding the biomechanical properties of the repaired cartilage. Implantation with the CHA scaffold matrix promoted cartilage repair and improved its compression capacity.

**Table 5:** Comparison of the levels of biomechanical properties between the groups (mean±SD)

Groups	Young modulus		Stress relaxation time		Creep time	
	6 months	12 months	6 months	12 months	6 months	12 months
CHA scaffold implantation group	0.48 ± 0.16	0.61 ± 0.17	1448 ± 337	1544 ± 407	1598 ± 314	2132 ± 213
Control group	0.35 ± 0.06	0.37 ± 0.13	1132 ± 257	1297 ± 70	1297 ± 339	1838 ± 262
Normal group	1.14 ± 0.16	1.23 ± 0.22	2222 ± 217	2209 ± 461	2504 ± 151	2738 ± 237
Statistic value	49.54	25.32	20.82	6.97	25.04	14.90
*P‐*value	≤0.001	≤0.001	≤0.001	0.015	≤0.001	0.001

CHA, cross‐linked sodium hyaluronate

### *Type II Collagen Expression*

Type II collagen content is an indicator for cartilage repair. Therefore, type II collagen expression was determined in the repaired cartilage. There were differences in type II collagen expression in repaired cartilage among the groups (Table 6). The type II collagen level in the CHA scaffold implantation group tended to be higher than that in the control group at 6 months (2.33 ± 1.50 *vs* 1.68 ± 0.56, *P* = 0.143) and 12 months postsurgery (3.37 ± 1.70 *vs* 2.06 ± 0.63, *P* = 0.108). The values in both groups at 6 months were significantly lower than normal cartilage at 6 months (4.37 ± 1.73, *P* = 0.010), whereas there was no significant difference among the groups at 12 months postsurgery (*P* = 0.065). Our results suggested that CHA is beneficial for type II collagen accumulation in the cartilage tissue. However, the cartilage repair with CHA was still different from normal cartilage within a short time.

**Table 6:** Comparison of expression of type II collagen between the groups

Time (after surgery)	Normal group	Control group	CHA scaffold implantation group	*P*	*P′*
6 months	4.37 ± 1.73	1.68 ± 0.56	2.33 ± 1.50	0.010*	0.143
12 months	4.47 ± 2.15	2.06 ± 0.63	3.37 ± 1.70	0.065	0.108

*P′* refers to the *P*‐value for statistical analysis of the difference between the CHA scaffold implantation and the control group.

* *P*‐value of *P* < 0.05 was considered statistically significant.

CHA, cross‐linked sodium hyaluronate

### *Glycosaminoglycan Expression*

To make clear the level of repaired cartilage, glycosaminoglycan was evaluated. There were significant differences among the groups in GAG expression evaluated at 6 and 12 months after surgery (Table 7), indicating that GAG expression in repaired cartilage was lower than that of normal cartilage. In addition, the GAG content in the cartilage of the control group was significantly lower than that of the experimental group (2.17 ± 0.43 *vs* 3.64 ± 1.17, *P* = 0.002 at 6 months and 2.27 ± 0.38 *vs* 4.12 ± 1.02, *P* = 0.002 at 12 months). Furthermore, there was no significant difference in GAG expression between the CHA scaffold implantation and normal groups evaluated both at 6 and 12 months postsurgery. Therefore, the GAG content in repaired cartilage after CHA scaffold implantation almost reached the normal level (Table 7). This suggested that CHA stimulated GAG accumulation at the repair site, and GAG accumulation increased with time.

**Table 7:** Comparison of expression of GAG between the groups

Time (after surgery)	Normal group	Control group	CHA scaffold implantation group	*P*	*P′*	*P″*
6 months	4.67 ± 1.20	2.17 ± 0.43	3.64 ± 1.17	0.002*	0.026*	0.17
12 months	4.21 ± 1.02	2.27 ± 0.38	4.12 ± 1.02	0.002*	0.002*	0.88

*P′* refers to the *P*‐value for statistical analysis of the difference between CHA scaffold implantation and control group. *P″* refers to the *P*‐value for statistical analysis of the difference between the CHA scaffold implantation and the normal group.

* *P*‐value of *P* < 0.05 was considered statistically significant.

CHA, cross‐linked sodium hyaluronate; GAG, glycosaminoglycan

## Discussion

Because of its unique physicochemical properties and biological functions, HA and its derivatives and preparations have been widely used in the clinic, including for OA, ophthalmologic operation, prevention of surgical adhesion, and burn treatment^11^, ^12^. However, raw HA are easily distributed and degraded by native enzymes and free radicals in bodily tissues, preventing their application in tissue engineering. Therefore, a number of CHA produced with different cross‐linkers and process parameters have been developed^13^, ^14^. The CHA with BDDE as the cross‐linker has proved effective in certain clinical trials^15^, ^16^. However, the safety and efficacy of CHA in tissue engineering, to date, have not been clarified.

In the present study, we evaluated the efficacy of CHA in cartilage repair in an animal microfracture model. We found that the surgery had little influence on the animals, and their behavior and gait were nearly normal, without any obvious clinical adverse reactions. The use of fibrin glue was not essential for the study; however, the results from our pre‐test showed that the fibrin glue maintained better stability. Therefore, we used the glue in our study. Furthermore, hematological examination showed that there was no significant difference between the CHA scaffold implantation and control groups, except for some biochemical ions and lipid factors. In addition, all the routine hematological factors tested in enrolled animals were within the normal physiological ranges. The above results confirmed that CHA scaffold implantation is safe and without any foreign‐body reaction or inflammation, which may induce implantation failure of scaffold materials^17^.

Cross‐linked sodium hyaluronate scaffold implantation improved the compressive ability of the repaired cartilage. The cartilage repair area under the naked eye and indentation test showed that the level of cartilage in the CHA scaffold implantation was greater than that of the control group (left knee) only operated with microfractures. CHA scaffold implantation slightly improved the compressive capacity of the repair cartilage. Furthermore, cartilage histopathology demonstrated that the surface of the cartilage repaired by CHA scaffold still showed fibrous tissue repair. Histopathological examination showed that the experimental and control groups displayed varying degrees of fibrous tissue repair on the surface of the damaged cartilage; safranin‐O staining of cartilage tissue sections showed that the white proteoglycan content of the two groups of repaired cartilage tissue was lower than that of the normal cartilage tissue. At 12 months postoperation, the distribution of cartilage proteoglycan in the experimental group was more extensive than that in the microfracture group, which supported the repair of the defective cartilage^18^.

A dense extracellular matrix is an important component of articular cartilage, which mainly contains type II collagen as well as proteoglycans^19^, ^20^. Furthermore, upregulation of type II collagen is one of the typical characteristics of chondrogenesis. Therefore, it is commonly considered that type II collagen and GAG reflect cartilage regeneration and are excellent predictors for the mechanical property. While the gross appearance and histopathology are two of most commonly reported results of cartilage repair, the histological score, GAG content and type II collagen composition are much better predictors for mechanical properties. The present study demonstrated that the CHA scaffold reduced injury‐induced cartilage degeneration and might promote cartilage defect repair ability according to the O'Driscoll Articular Cartilage Histology Score. Furthermore, CHA was beneficial for the accumulation of both type II collagen and GAG in the cartilage tissue, which increased with time, although the repaired cartilage was different from normal cartilage. The above findings indicated that CHA may be an attractive scaffold choice for cartilage tissue engineering^21^, which is in agreement with the results of Unterman. He investigated the efficacy of controlled HA presentation in synthetic–biologic composite materials as potential tissue engineering materials for the regeneration of cartilage and soft tissues. This study confirmed that HA‐binding hydrogels induced higher GAG and type II collagen production *in vitro* and induced modestly greater safranin‐O staining in repair cartilage *in vivo*. Other studies have indicated that HA has the potential to support upregulation of chondrocyte‐specific genes and produce cartilage‐like matrix rich in type II collagen and aggrecan^22^, ^23^. These findings are consistent with our results, which indicated that the CHA scaffold may help to promote the repair of defective cartilage, and it is, thus, a promising material in tissue engineering.

A limitation of the present study is that the number of experimental animals was small. Therefore, further animal study is needed.

In summary, the results of the effect of the CHA scaffold implant on cartilage repair show that it may help to promote the repair of defective cartilage safely. Implantation of a CHA scaffold could improve the compressive capacity in repairing cartilage. In conclusion, the present experimental results preliminarily demonstrated that the implantation of a CHA scaffold during microfracture is beneficial for cartilage repair and the reduction of injury‐induced degenerative cartilage changes. However, further animal study is needed to confirm the effect of CHA on cartilage repair.

## Disclosure

All the authors have no potential conflicts of interest, including financial interests, relationships, and affiliations relevant to the subject of the manuscript.

## References

[1] Laurent TC , Fraser JR . Hyaluronan. FASEB J, 1992, 6: 2397–2404. 1563592

[2] Hoffman AS . Hydrogels for biomedical applications. Adv Drug Deliv Rev, 2002, 54: 3–12. 11755703 10.1016/s0169-409x(01)00239-3

[3] Responte DJ , Natoli RM , Athanasiou KA . Identification of potential biophysical and molecular signalling mechanisms underlying hyaluronic acid enhancement of cartilage formation. J R Soc Interface, 2012, 8: 3564–3573. 10.1098/rsif.2012.0399 PMC3481566 22809846

[4] Buck DW II , Alam M , Kim JYS . Injectable facial fillers In: Erian A, Shiffman MA, eds. Advanced Surgical Facial Rejuvenation. German: Springer, 2012; 211–218.

[5] Dover JS , Rubin MG , Bhatia AC . Review of the efficacy, durability and safety data of two nonanimal stabilized hyaluronic acid fillers from a prospective, randomized, comparative, multicenter study. Dermatol Surg, 2009, 35: 322–330. 19207321 10.1111/j.1524-4725.2008.01060.x

[6] Cianflocco AJ . Viscosupplementation in patients with osteoarthritis of the knee. Postgrad Med, 2013, 125: 97–105. 23391675 10.3810/pgm.2013.01.2618

[7] Chen QQ , Chen JY , Zhang JQ , Wang Q , Ling PX . Process optimization of cross‐linked sodium hyaluronate scafold and its anti‐enzymatic degradation in vitro. Mater Rev, 2014, 28: 86–96.

[8] Schneider U , Schmidt‐Rohlfing B , Gavenis K , Maus U , Mueller‐Rath R , Andereya S . A comparative study of 3 different cartilage repair techniques. Knee Surg Sports Traumatol Arthrosc, 2011, 19: 2145–2152. 21409471 10.1007/s00167-011-1460-x

[9] Orth P , Zurakowski D , Wincheringer D , Madry H . Reliability, reproducibility, and validation of five major histological scoring systems for experimental articular cartilage repair in the rabbit model. Tissue Eng Part C Methods, 2012, 18: 329–339. 22081995 10.1089/ten.TEC.2011.0462

[10] Qing‐Hua Y , Yu‐Peng S , Haiyue J , Hong‐Xing Z . The significance of the biomechanical properties of costal cartilage in the timing of ear reconstruction surgery. J Plast Reconstr Aesthet Surg, 2011, 64: 742–746. 21130718 10.1016/j.bjps.2010.10.020

[11] Wang CT , Lin YT , Chiang BL , Lin YH , Hou SM . High molecular weight hyaluronic acid down‐regulates the gene expression of osteoarthritis‐associated cytokines and enzymes in fibroblast‐like synoviocytes from patients with early osteoarthritis. Osteoarthr Cartil, 2006, 14: 1237–1247. 16806998 10.1016/j.joca.2006.05.009

[12] Homandberg GA , Ummadi V , Kang H . High molecular weight hyaluronan promotes repair oflL‐l beta‐damaged cartilage explants from both young and old bovines. Osteoarthr Cartil, 2003, 11: 177–186. 12623289 10.1016/s1063-4584(02)00371-0

[13] Kenne L , Gohil S , Nilsson EM , *et al* Modification and cross‐linking parameters in hyaluronic acid hydrogels‐definitions and analytical methods. Carbohydr Polym, 2013, 91: 410–418. 23044151 10.1016/j.carbpol.2012.08.066

[14] Zhang R , Ma PX . Poly (alpha‐hydroxyl acids) / hydroxyapatite porous composites for bone‐tissue engineering. I. Preparation and morphology. J Biomed Mater Res, 1999, 15: 446–455. 10.1002/(sici)1097-4636(19990315)44:4<446::aid-jbm11>3.0.co;2-f 10397949

[15] Zhang Q , Lu H , Kawazoe N , Chen G . Pore size effect of collagen scaffolds on cartilage regeneration. Acta Biomater, 2005, 10: 2005–2013. 10.1016/j.actbio.2013.12.042 24384122

[16] Karageorgiou V , Kaplan D . Porosity of 3D biomaterial scaffolds and osteogenesis. Biomaterials, 2005, 16: 5474–5491. 10.1016/j.biomaterials.2005.02.002 15860204

[17] Zhang XO , Lv Y , Mao H , Fan XY , Huang SL , Guo XP . Bloomage Freda biopharm co.,ltd. hyaluronic acid scaffoIds: application research and product prospects. Chin J Tissue Eng Res, 2018, 22: 294–302.

[18] Lien SM , Ko LY , Huang TJ . Effect of pore size on ECM secretion and cell growth in Gelatin scaffold for articular cartilage tissue engineering. Acta Biomater, 2009, 5: 670–679. 18951858 10.1016/j.actbio.2008.09.020

[19] Bobick BE , Chen FH , Le AM , Tuan RS . Regulation of the chondrogenic phenotype in culture. Birth Defects Res C Embryo Today, 2009, 87: 351–371. 19960542 10.1002/bdrc.20167

[20] Bitter T , Muir HM . A modified uronic acid carbarbazole reation. Anal Biochem, 1962, 4: 330–334. 13971270 10.1016/0003-2697(62)90095-7

[21] Liu SY , Chen JY , Chen QQ , *et al* Construction of tissue‐engineered cartilage with cross‐linked sodium hyaluronate as scaffold materials in vitro. Chin J Tissue Eng Res, 2014, 18: 1191–1197.

[22] Chung C , Burdick JA . Influence of three‐dimensional hyaluronic acid microenvironments on mesenchymal stem cell chondrogenesis. Tissue Eng Part A, 2009, 15: 243–254. 19193129 10.1089/ten.tea.2008.0067 PMC2678568

[23] Erickson IE , Huang AH , Sengupta S , Kestle S , Burdick JA , Mauck RL . Macromer density influences mesenchymal stem cell chondrogenesis and maturation in photocrosslinked hyaluronic acid hydrogels. Osteoarthr Cartil, 2009, 17: 1639–1648. 19631307 10.1016/j.joca.2009.07.003 PMC2787866

